# Rapid Campimetry—A Novel Screening Method for Glaucoma Diagnosis

**DOI:** 10.3390/jcm11082156

**Published:** 2022-04-12

**Authors:** Fabian Müller, Khaldoon O. Al-Nosairy, Francie H. Kramer, Christian Meltendorf, Nidele Djouoma, Hagen Thieme, Michael B. Hoffmann, Friedrich Hoffmann

**Affiliations:** 1 H & M Medical Solutions GmbH, 14195 Berlin, Germany; 2 Ophthalmology Department, Faculty of Medicine, Otto-von-Guericke University, 39120 Magdeburg, Germany; 3 Department of Optometry, Berlin University of Applied Sciences and Technology, 10785 Berlin, Germany; 4 Center for Behavioral Brain Sciences, 39118 Magdeburg, Germany; 5 Ophthalmology Department, Charité—Universitätsmedizin Berlin, 12203 Berlin, Germany

**Keywords:** automated static perimetry, rapid campimetry, glaucoma, arcuate scotoma, visual field defect, telemedicine

## Abstract

One of the most important functions of the retina—the enabling of perception of fast movements—is largely suppressed in standard automated perimetry (SAP) and kinetic perimetry (Goldmann) due to slow motion and low contrast between test points and environment. Rapid campimetry integrates fast motion (=10°/4.7 s at 40 cm patient–monitor distance) and high contrast into the visual field (VF) examination in order to facilitate the detection of absolute scotomas. A bright test point moves on a dark background through the central 10° VF. Depending on the distance to the fixation point, the test point automatically changes diameter (≈0.16° to ≈0.39°). This method was compared to SAP (10-2 program) for six subjects with glaucoma. Rapid campimetry proved to be comparable and possibly better than 10-2 SAP in identifying macular arcuate scotomas. In four subjects, rapid campimetry detected a narrow arcuate absolute scotoma corresponding to the nerve fiber course, which was not identified as such with SAP. Rapid campimetry promises a fast screening method for the detection of absolute scotomas in the central 10° visual field, with a potential for cloud technologies and telemedical applications. Our proof-of-concept study motivates systematic testing of this novel method in a larger cohort.

## 1. Introduction

Peter Piot, Belgian virologist, director of the London School of Hygiene and Tropical Medicine, and COVID-19 advisor to the EU Commission, himself became seriously ill with COVID-19 in mid-March 2020. Since then, the scientific expert on viral diseases has called himself an expert by experience, indicating his new perspectives on viral diseases. New perspectives often enable new insights and promote possible solutions. One of us (F.H.), an ophthalmologist, has recently been diagnosed with normal tension glaucoma, and here, too, the new perspective of an experienced expert could support the development of a new examination method.

Glaucoma is one of the most common causes of irreversible blindness worldwide [1]. It is characterized by progressive optic neuropathy and loss of retinal ganglion cells (RGC) and is associated with visual field (VF) defects. Several approaches are available that allow for reproducible assessment of functional vision loss [2]. Among these, standard automated perimetry (SAP) is a common standard subjective visual field test, but it has limitations, such as response variability [3]. In fact, there have been many recent developments in the field of VF testing in glaucoma and its utility in clinical practice, such as “portable brain-computer interface” [4] or “fundus-tracked perimetry” [5]. Recent evidence from functional and structural testing [6,7] indicates that the macula is affected at early stages of glaucoma. This suggests the importance of central visual field testing, e.g., the 10-2 SAP testing algorithm, for the earlier detection of central VF damage besides its pivotal association with quality of life in affected individuals [8,9,10]. This motivates further studies to provide better evidence-based guidelines for testing the central 10–20° of the visual field.

Conventional perimetry employed in testing VF limits the detectability of early VF defects in glaucoma and might not be optimal to aid in the salvaging of retinal ganglion cells (RGCs) from permanent damage. Early histological studies revealed that 20–40% of RGCs are lost prior to any detectable VF defects on conventional perimetry [11]. Several psychophysical techniques have been adopted, aiming to spot glaucoma damage at its earliest stages, including tests employing motion perception. Although not widely adopted, several studies have indicated abnormal motion perimetry in glaucoma [12,13], even at early stages, i.e., ocular hypertension [14,15].

**Figure 1:**
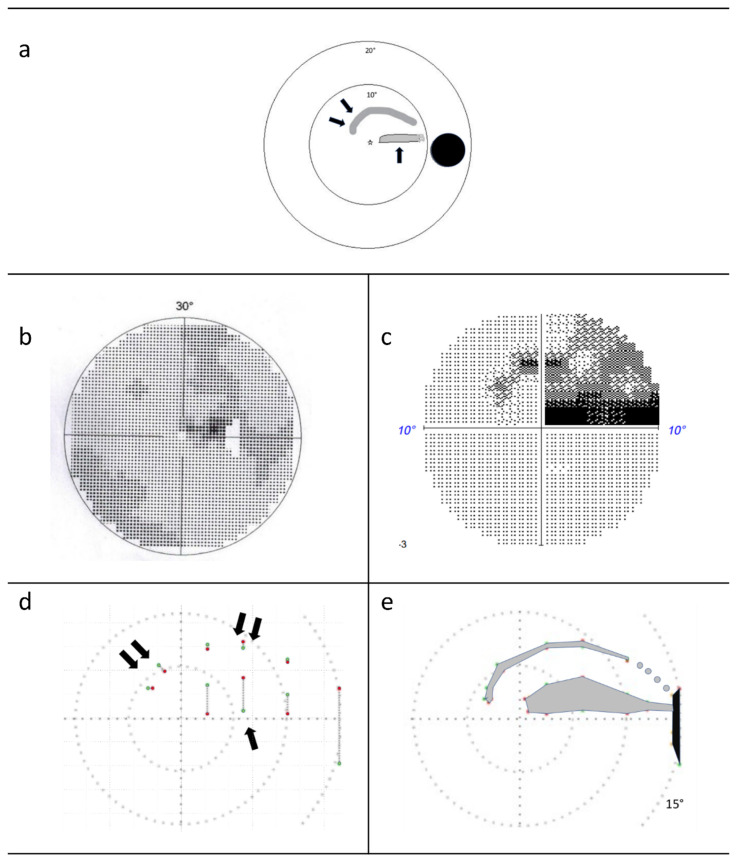
F.H. Visual field findings. (**a**) Sketch of the grey, drawn scotoma in the visual field of the right eye subjectively perceived by F.H. (first scotoma—one arrow, second scotoma—two arrows). (**b**) 30° field of view examined with Octopus 301. The scotoma is dark grey in the temporal upper field of view, the blind spot is shown in white. (**c**) 10° central visual field examined with the Humphrey Field Analyzer (HFA3). The absolute scotoma is black in the temporal superior visual field, and the relative scotoma is dark grey in the temporal and nasal superior visual fields. (**d**) Red and green dots, connected by a grey line, represent the beginning and end of the scotoma in the paramacular visual field after the screening procedure. One arrow marks the first scotoma, two arrows marks the second. (**e**) The 15° central visual field findings of scotoma delineation campimetry. After finding the two scotomas in the screening procedure, the exact scotoma borders were determined. The four grey spots between the arcuate scotoma and blind spot (black) represent the presumed scotoma course. In this area, the test point is thicker than the narrow scotoma and therefore does not become invisible. When the test dot moves quickly, a brightness difference is perceived here, indicating the defect.

In the present work, the perspective of an experienced expert (F.H.) served to explore a novel examination method for better understanding and earlier detection of VF defects based on the following case observation: In March 2017, F.H. observed a visual field defect on his right eye while rubbing the left eye. At the desk, a scotoma was identified as lying within the central 10° of the VF and was established as an arcuate scotoma in the superior temporal visual field, in analogy to locating the blind spot with a moving coin while fixating at a central point (Figure 1a). In June 2019, a second arcuate scotoma became apparent in the same eye (Figure 1a), it was unnoticed by Octopus 301 SAP (30°, Figure 1b) and was confirmed by Humphrey Field Analyzer (HFA3) 10-2 testing, applying 68 test points in the central 10° visual field (Figure 1c). Attempts to make the perceived visual field loss subjectively more salient utilized the observation that a small light, travelling rapidly through the visual field defect, was perceived as interrupted in the area of the scotoma. This insight was translated into the VF-testing method, i.e., rapid campimetry, which is described in the present paper. As proof-of-concept, it was applied in an additional five subjects with advanced glaucoma-related visual field defects.

## 2. Materials and Methods

### 2.1. Subjects

Five glaucoma patients (see Table 1 for demographic data), besides F.H., with central visual field defects were enrolled in this proof-of-concept study, which followed the tenets of the Helsinki Declaration and was approved by the local university hospital, after giving written informed consent. All subjects already had established glaucoma and met the inclusion criteria for open-angle glaucoma (n = 6, age 50 years or older) with an open anterior chamber and typical glaucomatous optic disc damage defined by a vertical cup ratio ≥ 0.7, retinal nerve fibre layer defect or localized rim depression, and glaucomatous visual field defects [16].

### 2.2. Standard Automated Perimetry Check (SAP)

Visual field defects were assessed using the 10-2 standard algorithm (subjects 1, 3, 4, 5 and F.H.) or 10-2 SITA Fast (subject 2) of the Humphrey Field Analyzer 3 (Carl Zeiss Meditec AG, Jena, Germany). The test stimulus was 4 mm^2^ in size (equivalent to a size III Goldmann stimulus, i.e., 0.43°) and presented for 0.2 s.

### 2.3. Rapid Campimetry

**Figure 2:**
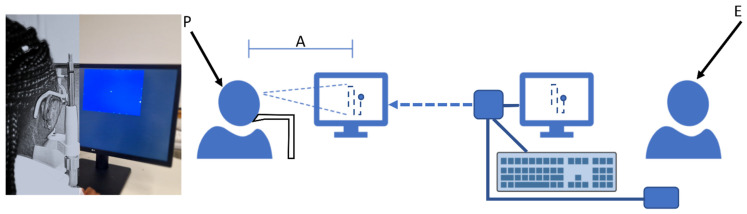
Rapid campimetry testing environment. Left Panel: Snapshot of the actual campimetry setting (with increased room lighting for better visualisation) with a volunteer fixating the centre of the testing area; left part of the image is masked to disable identification. Right panel: a sketch showing a person (P) looking at the monitor with a 40 cm distance (A) while an examiner (E) controls and runs the test on a different monitor.

Following the observation that a small light passed rapidly through the visual field defect is perceived as interrupted in the area of the defect, the central 10° visual field is tested in rapid campimetry with a bright test dot (140 cd/m^2^) on a dark screen (0.8 cd/m^2^) at a viewing distance of 40 cm (Figure 2). The visual field of the campimetry is extended temporally to 15° adjacent to the area of the blind spot to ensure that the patient understands the principle of the test by signalling the disappearance of the dot in the area of the blind spot. In the centre of the screen, there is a clearly visible cross as a fixation target (1.39° diameter) with lower brightness than the test marker.

The size of the test point was chosen to be as small as possible, such that it would not overlap the scotoma, while having good visibility at the same time. Because of the decreasing resolution from the centre to the periphery, the size of the test point increased with increasing distance from the fixation target. The optimal test point size was determined subjectively in pilot experiments (Table 2) and was 1.05 mm (0.16°) near the fixation point at a distance of 40 cm between the subject and the screen, and increased by 0.11 mm per degree, such that it had a size of 2.72 mm (0.39°) in the blind spot region. As the test point moves vertically, diagonally, and horizontally through the visual field, the size of the test spot changes automatically depending on the distance from the fixation point.

**Figure 3:**
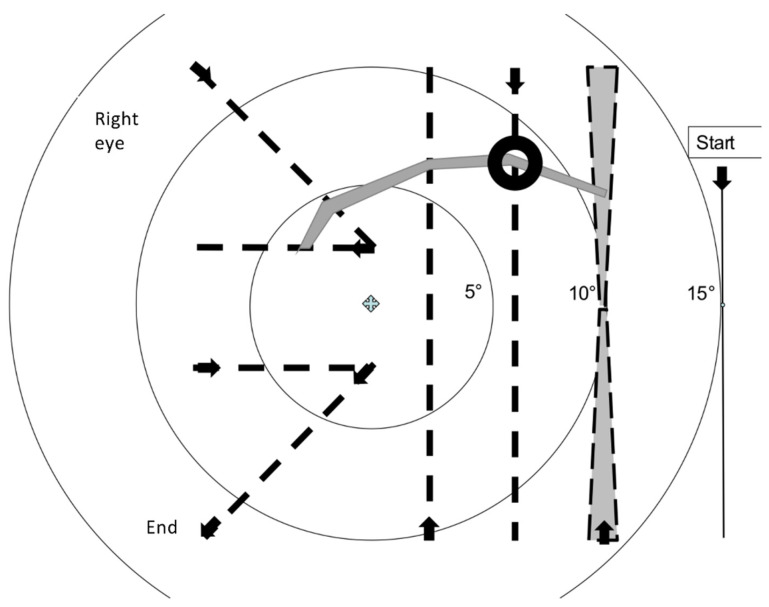
The path tested in the screening procedure of rapid campimetry is shown in dashed lines. In the arc scotoma marked by a black ring, 8° from the fixation point, the optimum test point speed was determined. The test point changes its diameter with the distance from the fixation point; the greater the distance, the greater its diameter. In the outer of the three vertical test lines, the used size change is overdrawn. If one wants to determine the examined area in this area, then the area is calculated as the sum of two identical trapezoids.

The most important difference in rapid campimetry in comparison to other visual-field testing methods is the running speed of the test point. The optimal running speed was determined subjectively on a narrow scotoma at a point approximately 8° from the fixation point, marked by a black ring (Figure 3). Different running speeds ranging from 0.18 cm/s to 24 cm/s were subjectively tested and the optimal speed was selected with which the scotoma was most reliably identified.

Using a too fast, 24 cm/s, or too slow, 0.18 cm/s, speed to run the test point disables the detection of the scotoma. Subjectively judged, the optimal speed of the test point seems to be ≈3 cm/s at 40 cm viewing distance from the screen. Here, it can be overlooked that the flat examination surface of the monitor results in an outward slowing of the velocity, since this variance at 10° results in about a 4% difference in velocity between the flat and curved surfaces. If the test point travels through the field of view at this speed along the seven vertical, diagonal, and horizontal paths mentioned in Figure 3, for a total length of ≈70 cm, then the test run passes through >1000 pixels (“test points”), depending on the resolution of the monitor (dpi). The specific screen area tested in rapid campimetry was 21.4 cm (442 pixels horizontally) by 14.1 cm (295 pixels vertically) and thus, for the test point progression of rapid campimetry (see below), ≈1400 test points. Due to the very fast update of the test point on the monitor (60 Hz), a subject perceives an uninterrupted line of light, that is, a point moving on the examination field of the monitor without interruption. The examination is completed within less than 30 s and the presence of absolute scotomas in the central 10° visual field can be largely excluded, if subjects see the test point uninterruptedly during the examination run.

The test point trajectory is, in principle, arbitrary. However, for better comparability of the results for “rapid campimetry”, a certain pattern is specified for the test point course. Within less than a minute, the test point first runs at 15° through the blind spot, then on three vertical, two diagonal and two horizontal lines through the central 10° visual field (Figure 3). The pattern of this test point course was chosen to follow the nerve fibre course traversing arcuate scotomas as perpendicularly as possible. As Aulhorn wrote, this is the best way to accurately determine glaucomatous scotoma boundaries [17].

The testing screen is coupled with an observation screen to enable monitoring of the test point by the examiner during examination. If the subject signals the disappearance or reappearance of the test point, these points of the scotoma rim are marked and the coordinates of these points are stored. In the examination result, the two points (scotoma start and end) are connected by a grey line symbolizing the scotoma, as shown in Figure 1d.

At the end of the test session, the examiner recognizes the suspected scotoma at the marked points at which the test point became invisible (off points) or visible again (on points). The scotoma can subsequently be delineated as in ordinary kinetic perimetry (“scotoma delineation campimetry”; duration approximately 1–10 min for one eye depending on the size of the VF defects) by moving the test point vertically, as, for example, shown in Figure 1e. Identifying the scotoma boundary accurately is facilitated by reducing the running speed of the test point, e.g., by a factor of 4 or 8.

If the examined area of each test point run is to be determined and set in relation to the square visual field with the horizontal and vertical diameter of the 10° area, and if the edge length of this square is 14.1 cm, then the total area to be examined is 198.81 cm^2^. The path tested in the screening procedure is shown in dashed lines in Figure 3. The test point changes its diameter with distance from the fixation point; the greater the distance from the fixation point, the greater its diameter. In the outer of the three vertical test lines, the size change used is exaggerated for clarity. The examined area is calculated approximately as the sum of two identical trapezoids, which are shifted vertically. Minimal deviations result from the fact that the test point change is linear only for lines running directly from the fixed point. The two trapezoids therefore have very slightly curved lines in the direction of travel. If they are placed next to each other, they approximately form a rectangle with half the running distance of the test point and the sum of the largest diameter of the test point at the top and the smallest diameter in the middle of the path. The area tested in rapid campimetry then adds up to a total of 13.41 cm^2^ of the total 198.81 cm^2^ from the three vertical, two identical diagonal, and two identical horizontal paths of the test point, and thus 6.75% of the paracentral visual field to be examined (Table 3).

## 3. Results

The case observation of F.H.’s scotomas is shown in Figure 1. The novel method of rapid campimetry verified the two subjectively observed scotomas. Figure 1d shows the result at the end of the test run of the rapid campimetry, and Figure 1e shows the result of the scotoma delineation campimetry. The red and green dots connected with a grey dotted line represent the scotoma’s start and end.

Five additional subjects (detailed in Methods) with a glaucomatous VF defect were included in this study to compare VF defects between SAP and rapid campimetry. Unintentionally, all five subjects had no SAP evidence of a VF defect in the fellow eye, which thus served as reference.

**Figure 4:**
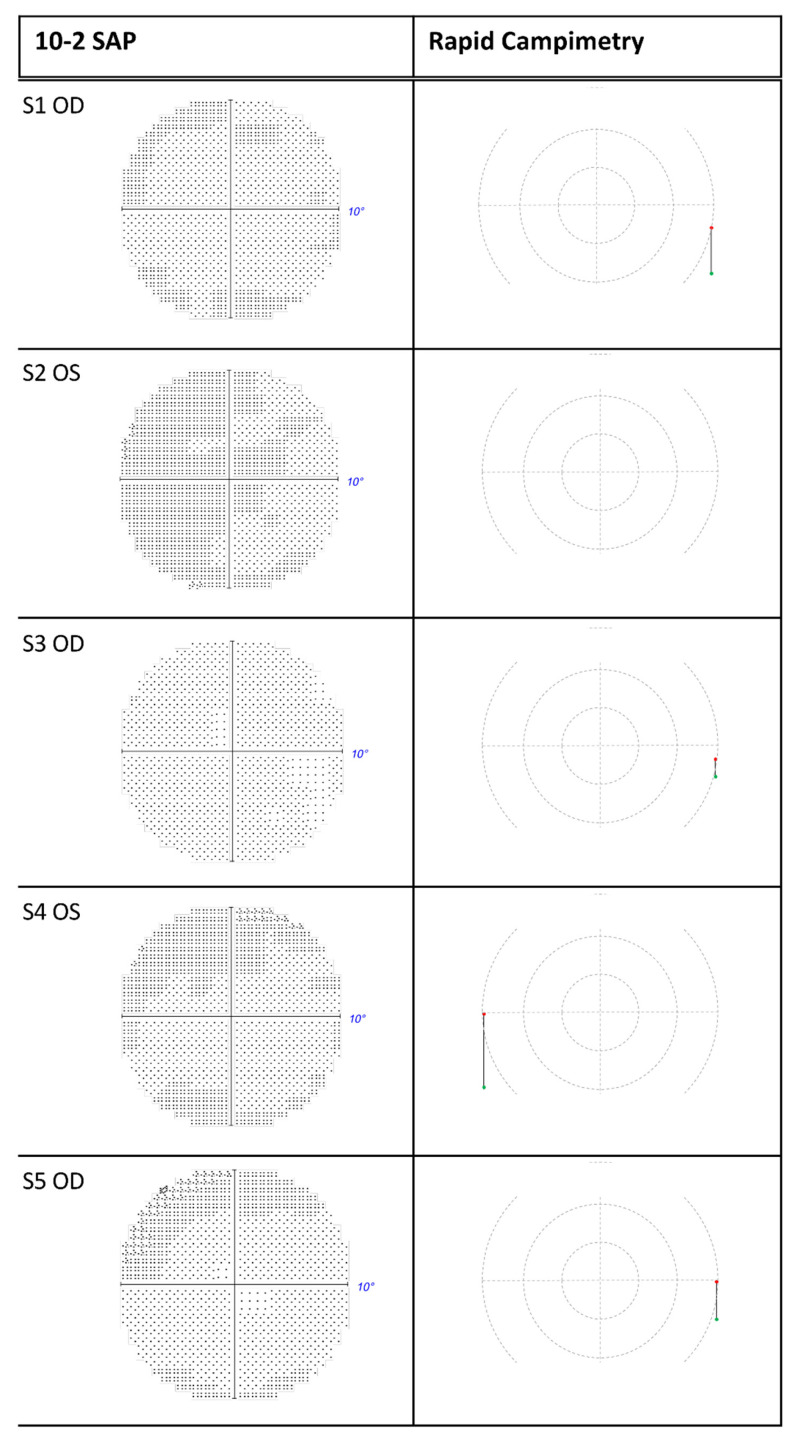
Eyes with normal visual field of subjects 1–5. The blind spot was detectable at 15° for all subjects except subject 2 (S2) with rapid campimetry. SAP = standard automated perimetry. OD = right eye; OS = left eye; S: subject; SAP: standard automated perimetry.

**Figure 5:**
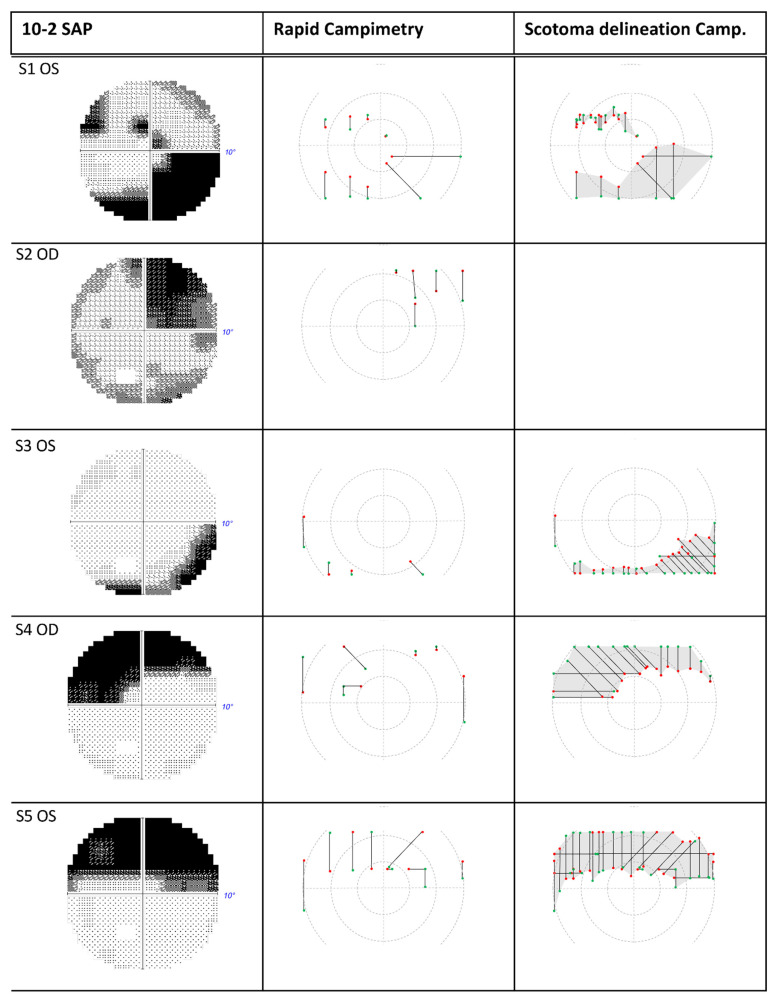
Eyes with visual field defects of subjects 1–5 compared between 10-2 SAP vs. rapid campimetry and scotoma delineation campimetry. SAP = standard automated perimetry. OD = right eye; OS = left eye; S: subject.

In general, there was an excellent agreement between rapid campimetry and SAP. All eyes without VF defects presented without abnormalities in either test (Figure 4). Similarly, the area and extent of the grey/black shaded regions of the VF defects in SAP corresponded to the scotoma line delineated by the rapid campimetry (Figure 5).

In combination with scotoma delineation campimetry, the following results are obtained for each subject compared to SAP: In subject 1, HFA detected scotomas in the upper visual field and normal sensitive retina between these scotomas. Scotoma delineation campimetry found instead a continuous arcuate scotoma in the same location. In subject 2, both HFA and campimetry demonstrated comparable findings showing an upper quadrant scotoma (Figure 5). In the lower visual hemifield of the left eye of subject 3, there was a relative scotoma in the centre of the arcuate scotoma, which scotoma delineation campimetry identified as an absolute scotoma. In subject 4, both campimetry and SAP depicted a similar arcuate scotoma in the superior VF of the right eye. Finally, subject 5 has an upper arcuate scotoma at a site of relative scotoma that rapid campimetry classified as an absolute scotoma.

## 4. Discussion

The aim of this proof-of-concept study was to compare the novel visual field examination technique, rapid campimetry, with the established standard automated perimetry (SAP) in a case series regarding the detection of glaucomatous defects. In this six-subject sample, we found strong agreement between SAP and rapid campimetry in identifying VF defects in all eyes.

### 4.1. Increasing Attention via Fast Stimulus Movement

In the established SAP, the response behavior of the examinees is strongly dependent on their attention, since they are supposed to judge the appearance of a test point just at the threshold of perception and in weak contrast with the surroundings. The image change occurs so weakly or slowly that it is easily overlooked, but it is necessary in this form to define the threshold of perception [18]. One of the most important functions of the retina, namely, enabling the perception of rapid movement, was important in evolution because detection of the movement of a prey animal or enemy provided a survival advantage [19]. The perception of fast motion, however, is not tested in threshold perimetry. Notably, in order to identify retinal areas without light perception, i.e., whether there are absolute scotomas, fast motion can be used in combination with high contrast, with several key advantages, such as hardly strained participant attention.

### 4.2. Proportion of the Examined Visual Field Area in the Paracentral Visual Field

Another important difference between the different VF testing methods is the portion of the visual field actually examined. Testing the central VF using either Octopus, G1 program (17 points), or HFA3, 10-2 program (68 points) and employing Goldmann point size III, i.e., 4 mm^2^ [4], the area examined only covers 3.1% and <1.0% of the central visual field, respectively. Here, at the examination distance of 30 cm, the 10° area of the central visual field is 87.58 cm^2^ with a radius of 5.28 cm with minimal error variability due to the spherical surface deviation. These values explain why the arcuate scotoma was not found in F.H. with the Octopus perimeter. More accurate results can be expected with rapid campimetry where 6.75% of the paracentral visual field is tested.

### 4.3. Accuracy of Rapid Campimetry

To further test the accuracy of rapid campimetry vs. other standard perimetry, i.e., HFA3, we assessed whether glaucomatous VF defects were comparable in both techniques. Here, the examination of glaucomatous VF defects of five participants demonstrated agreement in the findings. In addition, rapid campimetry appeared to detect scotomata areas that were missed in the standard HFA3 test. The findings of subjects 1, 3, and 5, as well as F.H. suggest a superiority of the rapid campimetry vs. HFA3: For example, for F.H., the HFA found a relative scotoma in the upper visual field, whereas the campimetry instead found an absolute narrow arcuate scotoma at the same location (Figure 1e). As shown in Table 2, the angular diameter of the test point at the edge of the central 10° field of view is 0.31° compared to the conventional perimeter test mark III with a diameter of 0.43° at any point in the VF [18]. This latter large test mark cannot totally disappear in the narrow, approximately 0.35°-wide scotoma of Figure 1e, and cannot be perceived as an absolute scotoma, but only as a relative one. Finding these narrow scotomas appears to be facilitated by rapid campimetry’s technique of a continuous vertical light line of ≈1400 closely spaced test points that overlap during motion and intersect all nerve fibres running to the blind spot.

### 4.4. Detection of Arc Scotomas

The method of rapid campimetry is similar to the campimetry described by Rönne and developed by his teacher Bjerrum, where a 1 cm-sized test point with angular diameter of 0.29° moves slowly on a black rod at 4 m^2^ square black wall at 1–2 m distance [20]. In rapid campimetry, the dimensions are reduced and tailored to today’s technology, as well as having the crucial feature of rapid movement. According to Rönne, the first early defects in glaucoma usually present in the Bjerrum area as small paramacular scotomas, which may be arcuately connected to the blind spot [20]. An explanation for arcuate scotomas is easily given by comparing the nerve fibre course in the retina with the shape and location of the arcuate scotomas where glaucoma damages individual optic nerve bundles and leads to interruption of the input from the corresponding retinal sites, leaving other bundles intact [21]. With today’s standard examination methods, arcuate scotomas are hardly detected as such early stages, although their presence is theoretically probable [21].

Recently, finer patterns than the standard 24-2 VF tests, e.g., a 6 × 6° grid, have been applied, and studies have confirmed that multiple macular VF defects can occur in glaucoma, of which arcuate scotoma is the most common [22,23]. These VF defects could also correspond to structural damage [24]. More recently, a new testing paradigm, the 24-2C, has been developed, in which 10 asymmetrically distributed test points from the 10-2 grid are integrated into the 24-2 grid so that both the central and peripheral visual fields can be tested. Nevertheless, testing the central 10 degrees supports higher resolution in terms of a detailed description of VF defects and better structure–function agreement [25].

### 4.5. Automation of Test Point Movement in the Paramacular Visual Field

Glaucoma is a group of progressive optic neuropathies characterized by degeneration of retinal ganglion cells [26]. The probable consequence of such ganglion cell degeneration is absolute rather than relative scotoma. Aulhorn, in reviewing 961 visual fields of glaucoma subjects, found that very early scotomas, despite their small extent, are usually absolute and very rarely relative [17]. In principle, the shape of a scotoma can be described well with kinetic perimetry, but small paramacular scotomas can be easily missed [17]. The requirement for slow test point movement can be met by the instrument only if large movement distances on the examiner’s side correspond to a small visual angle on the subject’s side. This is only possible, however, if very large-area examination screens are used for direct test point guidance, as for example with the Bjerrum wall, or if a translation mechanism is used for indirect test point movement [17].

The combination of the two demands may seem absurd, to increase the running speed of the test point for a safer scotoma detection on the one hand, and to move the test point as slowly as possible for an accurate definition of the scotoma margins on the other hand. However, both demands belong together, and only together do they fulfill their task perfectly. With the automatic test point movement, which can be slowed down by a factor of 4 or 8, the rapid campimetry meets Aulhorn’s demand of translation mechanics in the paramacular range. In this way, it is possible to translate the advantages of Bjerrum’s and Rönne’s campimetry [20] into a novel technique and to combine it with the attentional enhancement of the fast movement.

### 4.6. Limitations of the Study

Our case-series study has a number of limitations which need to be addressed in a follow-up study on a larger participant cohort. The study was designed to provide proof-of-concept of rapid campimetry and was not designed to assess the sensitivity and specificity of the approach. For the latter purpose, a systematic investigation with a greater sample size is essential, including patients with different disease states and healthy controls. Further, potentially confounding effects of visual pathologies, e.g., optic media opacities, deserve attention in future studies. Finally, the quality of the fixation and its relation to the campimetry outcome has not been addressed in the present study, where patients were instructed to fixate the central target during testing and repeatedly reminded of the importance of central fixation. Online tracking of eye movements and fixation monitoring would help to assess whether maintaining central fixation is an issue during rapid campimetry testing.

### 4.7. Outlook

In addition to glaucoma screening, there are a number of fields where rapid campimetry might be of value. One potential application of the new examination method leads back to the beginning of the text—the COVID-19 pandemic, which has alerted us to the importance of telemedicine. Rapid campimetry is enabled via the internet and leverages the potential of cloud technologies, as a commercially available computer connected to the internet enables rapid campimetry virtually anywhere in the world with very low barriers to entry compared to current investigative methods. Possibly, this novel method could also help correlate morphologic differences of certain scotomas with their cause through more accurate scotoma description: Lachenmayr, for example, points out that in addition to mechanical nerve fibre damage due to intraocular pressure, there is vascular damage with typically classic nerve fibre bundle defects that manifests as arcuate scotoma [18]. Furthermore, migraine is considered a risk factor for glaucoma [27], which raises the question of whether visual field defects associated with migraine aura can be morphologically distinguished from typical glaucoma-related scotomas.

### 4.8. Conclusions

Our present proof-of-concept study suggests that rapid campimetry has advantages in glaucoma-screening compared to SAP. However, follow-up assessments are needed that investigate greater sample sizes of patients and healthy controls to assess the value of rapid campimetry as a screening and diagnostic tool for VF defect detection in glaucoma. In short, this method appears to be comparable to standard perimetry in the detection of central VF defects in glaucoma, and holds promise of applicability in ophthalmology as a screening and telemedicine tool.

## 5. Patents

European patent pending: Nr. EP21151704.0, EP21176171.3, EP21196409.3—PCT/EP2022/050765 “Verfahren und Einrichtung zum Messen des Gesichtsfeldes einer Person”.

## Figures and Tables

**Table 1:** Demographic data of the subjects.

	Gender	Age [Years]	BCVA [logMAR] OD	BCVA [logMAR] OS	MD 10-2 OD [dB]	MD 10-2 OS [dB]
S1	m	81	0.0	0.2	0.29	−15.48
S2	f	80	0.80	0.4	−14.52 *	−1.73 *
S3	m	55	−0.1	−0.1	1.20	−3.64
S4	m	70	0.0	0.1	−10.77	−1.03
S5	f	62	0.0	0.0	−0.40	−13.30

S: Subject; m: male; f: female; BCVA: Best corrected visual acuity; OD: right eye; OS: left eye; logMAR: logarithm of minimal angle of resolution; MD: mean visual field deviation of 10-2 SITA standard VF; dB: Decibels; * 10-2 SITA fast protocol.

**Table 2:** Various test point sizes in relation to position.

Distance [°] From Fixation Point	Diameter [mm] of the Test Point	Angle Diameter [°] of the Test Point
0.0°	1.05	0.16°
1.0°	1.16	0.17°
2.5°	1.33	0.19°
5.0°	1.61	0.23°
7.5°	1.88	0.27°
10.0°	2.17	0.31°
12.5°	2.45	0.35°
15.0°	2.72	0.39°

**Table 3:** Area calculation of the tested visual field fractions during the test run.

	Greatest Test Point Thickness [cm]	Smallest Test Point Thickness [cm]	Sum of Both Test Point Thicknesses [cm]	Half the Running Distance of the Test Point [cm]	Area [cm^2^]
Vertical 1	0.26	0.22	0.48	7	3.35
Vertical 2	0.24	0.18	0.42	7	2.93
Vertical 3	0.22	0.13	0.35	7	2.47
Diagonal 1 + 2	0.24	0.14	0.38	7.44	2.81
Horizontal 1 + 2	0.19	0.14	0.33	5.6	1.85
Total					13.41

## Data Availability

Upon request.
